# Factors associated with research activity among radiologists: results of a Nordic survey

**DOI:** 10.1186/s13244-025-02108-0

**Published:** 2025-10-26

**Authors:** Pyry Jylhä-Vuorio, Irina Rinta-Kiikka, Anne Mäkikangas, Jussi Hirvonen, Tiina Luukkaala, Otso Arponen

**Affiliations:** 1 Department of Radiology, Kanta-Häme Central Hospital, Hämeenlinna, Finland; 2 Department of Radiology, Tampere University, Faculty of Medicine and Health Technology, and Tampere University Hospital, Tampere, Finland; 3 Work Research Centre, Faculty of Social Sciences, Tampere University, Tampere, Finland; 4 Health Sciences, Faculty of Social Sciences, Tampere University and Development and Innovation Centre, Tampere University Hospital, Tampere, Finland; 5 Institute of Clinical Medicine, School of Medicine, University of Eastern Finland, Kuopio, Finland

**Keywords:** Academic radiology, Physician-scientist, Barriers to research, Radiological research, Survey

## Abstract

**Background:**

Research activities often compete with clinical work and personal life for the time of physician-scientists. To overcome barriers to research, examining the factors affecting research productivity is important.

**Objectives:**

To identify potential personal, physician-dependent, and external physician-independent factors affecting researcher productivity in a cohort of Nordic radiologists.

**Methods:**

A prospective survey was open to responders from 10 May 2023 to 23 June 2023. The survey was distributed to radiologists and radiology residents in the Nordic countries (Denmark, Finland, Norway, and Sweden) through multiple channels. We collected demographic information, details about work and academic careers, and opinions and attitudes on work, research, and personal life using a Likert-scale questionnaire.

**Results:**

A total of 192 participants responded (mean age 46.4 (SD: 11.03), 88 (45.8%) males, 103 (53.6%) females). Of the 134 (69.8%) respondents who reported having made any past academic contribution, 88 (46.4%) indicated active research participation. Active researchers expressed more agreement that they have the skills (*p* < 0.001) and resources (*p* < 0.001) for research and are able to maintain expertise (*p* = 0.003). Responders most frequently reported that having time for research (*n* = 94/155, 60.6%), motivation (*n* = 56/155, 36.1%), more funding (*n* = 36/155, 23.2%), and a higher salary (*n* = 36/155, 23.2%) would increase research involvement.

**Conclusions:**

We identified several differences between radiologists who are active in research and those who are not. The participants identified time, financial means, and motivation as key factors that could increase research involvement.

**Critical relevance statement:**

Academic radiologists with active research careers report having the necessary skills and resources for research, teaching, and learning more frequently than radiologists less active in research.

**Key Points:**

- Active researchers are more in agreement with having the skills and resources for research.
- Active researchers expressed more agreement with interest in teaching, keeping expertise up to date, getting enough sleep, and being less distracted by social media.
- Researchers with a recent history of funding reported more publications. There may be research potential for physicians engaged in self-financed research.

**Graphical Abstract:**

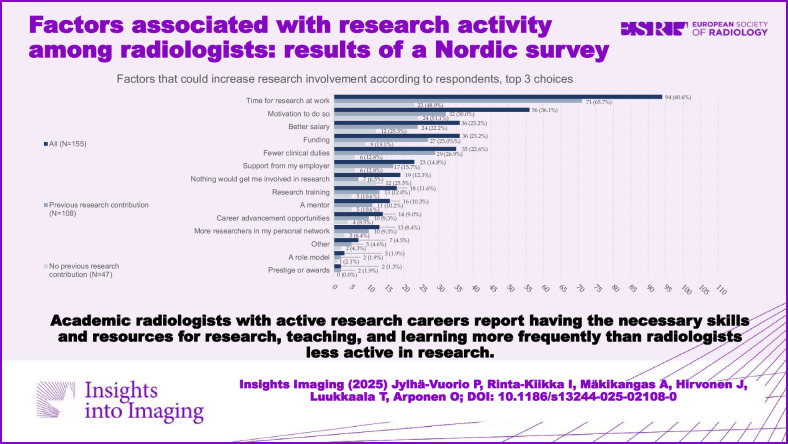

## Introduction

Radiology research is needed to keep pace with technological development, concomitant with the increasing pace of biomedical research [1]. The funding landscape can be unpredictably affected by external factors such as governmental policy, world events such as the COVID-19 pandemic, and economic cycles [2, 3]. For example, the COVID-19 pandemic imposed several challenges on academic research, including cancelled scientific meetings that limited collaboration, reduced lab access, delayed access to state-of-the-art equipment, hiring disruptions, and financial disruptions, with the full effects are still unfolding [3, 4]. In addition to other sources of uncertainty, clinical duties compete for academic radiologists’ time.

Academic radiologists are an integral part of the medical research community, but considering the uncertainties in available funding and time, there are concerns about the future appeal of conducting research. International, often resident-oriented questionnaires have aimed at identifying barriers to research in radiology [5, 6]. Many young physicians consider research important but do not commit to a career in research [7–10]. Previous studies have identified several physician-dependent and external factors that may affect the likelihood of conducting research [11–15], including personal interest, motivation, research funding, personal financial aspirations, work-life balance, time spent studying, interest in teaching, clinical workload, on-call duties, career plans, role models, mentoring, research competencies and availability of research-oriented programmes or training [6, 7, 16–21].

Much of the previous research on researcher productivity is focused on residents and other medical specialties. We hypothesise that radiologists face similar intrinsic and external factors that may affect the general likelihood of conducting research among physicians. To identify possible radiologist-dependent and external factors affecting the likelihood of conducting radiological research, we developed a survey aimed at radiologists and radiology residents of all career stages in the Nordic countries.

## Materials and methods

### Study overview

We sought to investigate different factors that could, based on previous literature, influence the research activity of Nordic radiologists. We designed a survey to probe relevant factors of interest and distributed it to Nordic radiologists of all career stages through multiple distribution channels in late spring 2023. Participation was based on informed consent. The study was approved by the Ethics Committee of the Tampere Region (study identifier: HT22004).

### Survey design

We aimed to collect relevant demographic information, self-reported academic experience, publication history, career and work information, and indicators of occupational well-being based on the existing literature. To achieve this, we developed an electronic questionnaire (Supplemental Materials a) [22, 23]. The consent and survey data entries were collected electronically using the Research Electronic Data Capture (REDCap) platform version 13.7.5. We consulted a survey design expert on the design of the questionnaire and a REDCap platform expert on coding the survey on the REDCap platform. The survey functionality and response time were pre-tested, and the response time was estimated to be 5–7 min. The estimated survey response time was then rounded up and advertised as 10 min. This was done to encourage complete responses, mitigate feelings of disengagement, and discourage possible repeat responses [24].

### Survey distribution

We conducted the study in the Nordic countries (Denmark, Finland, Norway, and Sweden) between 10 May 2023 and 23 June 2023. We targeted all radiologists and radiology residents within these countries that we could reach. Iceland does not have a national radiologist training programme and was therefore excluded. The survey was distributed through academic and professional networks. We approached representatives of medical unions and hospital and university administrators for help with the survey’s distribution. Between 11 May and 27 May, the radiological societies of Finland and Sweden published the survey on their website, the Danish Society of Radiology published the survey on Facebook, and the Norwegian Society of Radiology distributed the survey to their members through their membership mailing list. The survey was also physically promoted at a stand in the Nordic Congress of Radiology 2023 in Helsinki on 25–26 May, 2023. We also encouraged the survey participants to circulate the to relevant professional contacts who might not have otherwise been reached. Based on publicly available data, we estimate that, at most, approximately 5700 practicing radiologists could have been reached in our target countries, but because no single distribution channel exists that could reach all of these radiologists, the total reach of the survey in a multi-channel distribution approach cannot be reliably estimated. We estimate that in Finland there are 674 practicing radiologists, in Norway 1512, in Sweden 2820, and in Denmark 700 [25–28].

We utilised established effective methods to increase response rates when possible, such as advertising a pre-tested short survey completion time. We promoted the survey by having an incentive, by promising to make a charitable donation if the number of voters per country exceeded 300 respondents [29, 30].

### Inclusion process

To be included, a respondent had to give informed consent and at least partially respond to study-related questions. The least complete responses that could be utilised included responses at least until the question ‘Who has funded your research in the past 5 years’. The respondent had to be either a radiologist or a radiology resident from one of the Nordic countries (Denmark, Finland, Norway, or Sweden). The response also had to signify a unique responder. Participants who took the survey more than once were identified through their e-mail addresses, and only the most complete and most recent response was included.

### Definition of terminology

For the purposes of this study, respondents who indicated having any previous or current research experience were categorised as ‘active’ or ‘non-active’ researchers based on their current research involvement. We defined active researchers as respondents who indicated that they had some research experience and were currently involved in research either full-time, part-time, or in their free time. Non-active researchers were respondents who indicated having previous research experience without current research involvement.

### Statistical analysis

We analysed data with IBM SPSS Statistics (v. 23 for Windows, SPSS Inc.). Respondents’ demographic and baseline characteristics of the respondents are reported as medians and interquartile ranges or as a number of participants with percentages. Likert-scale distributions are expressed using means with standard deviations (SDs), as they describe distributions better than medians with interquartile ranges [31]. The differences in these distributions were tested using Welch’s *t*-test. All tests were two-sided, and *p*-values < 0.05 were considered statistically significant.

## Results

### Demographics

**Fig. 1:**
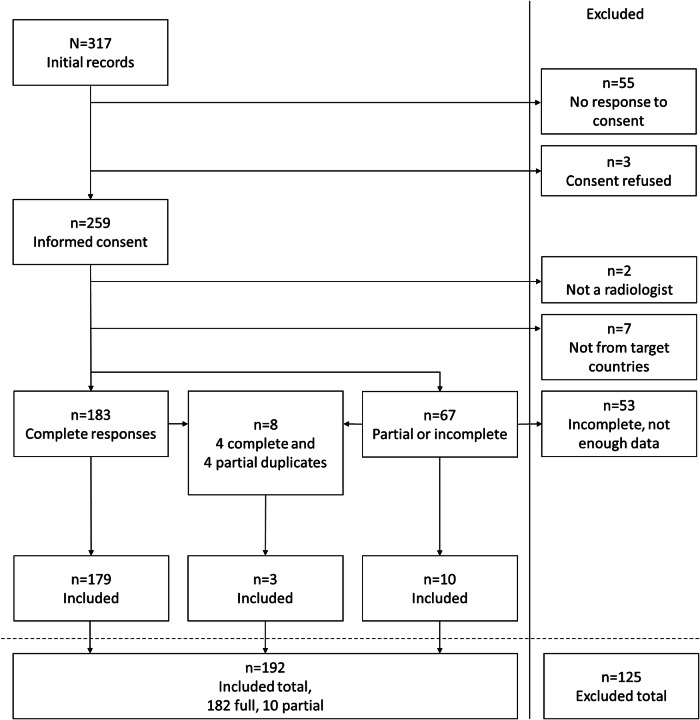
Flowchart describing the inclusion process

We received a total of 317 responses during the study period. Informed consent was provided in 259/317 (81.7%) cases; responders who declined to participate in the study or did not give informed consent (58/317 (18.3%) cases) were excluded. We further excluded 55/317 (17.4%) incomplete responses that could not be analysed, 7/317 (2.0%) responses from responders residing outside of the Nordic countries (Denmark, Finland, Norway, and Sweden), 2/317 (0.6%) responses from non-radiologist responders, and 5/317 (1.6%) duplicate responses. The inclusion process is illustrated in Fig. 1. After the exclusion of 125/317 (39.4%) responses, we included 192/317 (60.7%; 182/192 (94.8%) complete, and 10/192 (5.2%) incomplete) responses. The demographics of the cohort are outlined in Table 1.

**Table 1:** Study participant demographics

	All participants (*N* = 192)	Participants according to research experience		Current research work status of participants with research experience	
		Has research experience (*n* = 134)	No research experience (*n* = 58)	Active researcher (*n* = 87)	Has research experience, not an active researcher (*n* = 47)
Mean age (years), (SD)	46.5 (11.0)	47.3 (11.51)	44.7 (9.70)	46.9 (11.11)	48.0 (12.30)
Gender, *n* (%)					
Male	88 (45.8%)	62 (46.3%)	26 (44.8%)	39 (44.8%)	23 (48.9%)
Female	103 (53.6%)	71 (53.0%)	32 (55.2%)	47 (54%)	24 (51.1%)
Radiologists or residents, *n* (%)					
Radiologists	152 (79.2%)	111 (82.8%)	41 (70.7%)	72 (82.8%)	39 (83.0%)
Residents	40 (20.8%)	23 (17.2%)	17 (29.3%)	15 (17.2%)	8 (17.0%)
Country of residence, *n* (%)					
Denmark	39 (20.3%)	34 (25.4%)	5 (8.6%)	24 (27.6%)	10 (21.3%)
Finland	114 (59.4%)	72 (53.7%)	42 (72.4%)	45 (51.7%)	27 (57.4%)
Norway	22 (11.5%)	16 (11.9%)	6 (10.3%)	9 (10.3%)	7 (14.9%)
Sweden	17 (8.9%)	12 (9.0%)	5 (8.6%)	9 (10.3%)	3 (6.4%)
Years of experience in radiology (Median, Min–Max)	14 (1–55)	15 (1–55)	10.5 (1–34)	15 (1–55)	15 (1-48)
Employment status, *n* (%)					
Working full-time	159 (82.8%)	108 (80.6%)	51 (87.9%)	71 (81.6%)	37 (78.7%)
Working part-time	32 (16.7%)	25 (18.7%)	7 (12.1%)	15 (17.2%)	10 (21.3%)
Primary work sector, *n* (%)					
Public sector	172 (89.6%)	121 (90.3%)	51 (87.9%)	80 (92.0%)	41 (87.2%)
Government (local or national)	6 (3.1%)	4 (3%)	2 (3.4%)	2 (2.3%)	2 (4.3%)
Private	12 (6.3%)	4 (3%)	5 (8.6%)	4 (4.6%)	3 (6.4%)
Charity, trust, or equivalent, or other response	2 (1.0%)	19 (14.2%)	0 (0.0%)	1 (1.1%)	1 (2.1%)
Research work status, *n* (%)					
Doing research full-time^a^	3 (1.6%)	3 (2.2%)	0 (0.0%)	3 (3.4%)	N/A
Doing part-time research^a^	29 (15.1%)	29 (21.6%)	0 (0.0%)	29 (33.3%)	N/A
Doing research mainly in free-time^a^	57 (29.7%)	55 (41.0%)	2 (3.4%)	55 (63.2%)	N/A
Not doing research	103 (53.6%)	47 (35.1%)	56 (96.6%)	N/A	47 (100.0%)
Civil Status, if disclosed, *n* (%)					
Married, civil union, or committed relationship	165 (85.9%)	117 (87.3%)	48 (82.8%)	80 (92.0%)	37 (78.7%)
Single, divorced, or widowed	24 (12.5%)	14 (10.4%)	10 (17.2%)	5 (5.7%)	9 (19.1%)
Has children, *n* (%)					
Yes	147 (76.6%)	107 (79.9%)	40 (69.0%)	69 (79.3%)	38 (80.9%)
No	45 (23.4%)	27 (20.1%)	18 (31.0%)	18 (20.7%)	9 (19.1%)

*N/A* not applicable

^a^ Defined as “active researcher”

### Research background of the respondents

Of the 189/192 (98.4%) respondents who had listed their academic titles, 54/189 (28.6%) had at least a Ph.D. Of these, 18/54 (33.3%) had finished a post-doctoral programme, while 4/54 (2.1%) participated in one. A total of 10 (5.3%) of the 189 were professors, and 29 (15.3%) were assistant professors or equivalent (e.g., docents).

**Fig. 2:**
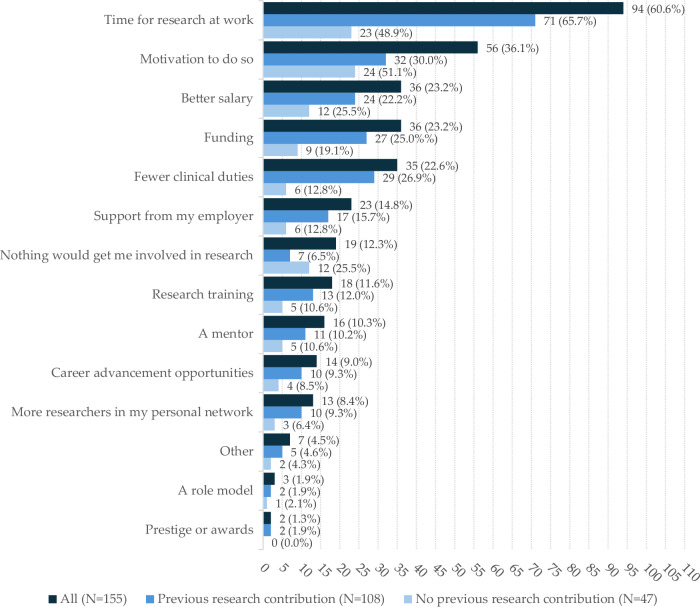
Factors that could increase research involvement according to respondents. Up to three choices were allowed; responses not following the guideline were excluded. Choices are reported by number and percentage per group

Altogether, 134/192 (69.8%) participants indicated having some research experience, and 87/134 (64.9%) identified as active researchers. The median reported research experience of participants with some research experience was seven years (interquartile range 13.75). Of those with some research experience, 99/134 (73.9%) indicated having had experience working as part of a research group, 26/134 (19.4%) indicated having worked as a supervisor, and 20/134 (14.9%) indicated they had been consulted by a research group without direct participation. Every fifth (26/134, 19.4%) participant with some research experience indicated having contributed to research but had no published articles or articles in review. Of the respondents with some research experience, 91/134 (73.1%) and 96/134 (71.6%) indicated that they had received funding for research within the previous 1 year and 5 years, respectively. We asked all respondents to choose the top three factors that could increase their research involvement, responses were grouped for those with a research background, and those without one (Fig. 2).

### Attitudes and opinions on clinical work, research, and teaching among active researchers and others

Differences in attitudes and opinions on clinical work, research, and teaching among active and non-active researchers and those without research experience are presented in Tables 2 and 3. Active researchers expressed higher agreement with several clinical work, research, and teaching-oriented claims in comparison to non-active researchers and those without research experience: Active researchers considered research important for their career (mean 3.62 (standard deviation: 1.07) vs. 2.19 (SD: 1.04), *p* < 0.001), were inspired by colleagues who do research (4.10 (SD: 0.80) vs. 3.05 (SD: 1.11), *p* < 0.001), wanted to conduct medical research (4.01 (SD: 1.10) vs. 2.28 (SD: 1.11), *p* < 0.001), thought they had the necessary skills to conduct research (3.96 (SD: 1.01) vs. 2.80 (SD: 0.99), *p* < 0.001), had access to funding (3.33 (SD: 0.88) vs. 2.73 (SD: 1.03), *p* < 0.001) and training (3.26 (SD: 1.02) vs. 2.71 (SD: 0.99), *p* = 0.001), felt they had access to research collaborations (3.38 (SD: 1.21) vs. 2.73 (SD: 1.15), *p* < 0.001], felt being able to keep expertise up to date (3.95 (SD: 0.93) vs. 3.55 (SD: 0.90), *p* = 0.003), were interested in teaching (4.27 (SD: 0.82) vs. 3.45 (SD: 0.96), *p* < 0.001), and had been positively affected by a mentor or a role model (3.85 (SD: 0.97) vs. 3.21 (SD: 1.11), *p* < 0.001). They also expressed more often getting enough sleep (3.20 (SD: 1.09) vs. 2.76 (SD: 1.09) *p*  = 0.008). On the other hand, active researchers expressed lower agreement regarding not being suited for academic research (2.11 (SD: 1.10) vs. 3.00 (SD: 1.08), *p* < 0.001), not getting along with academic people (1.69 (SD: 1.00) vs. 1.97 (SD: 0.84), *p* = 0.05) as well as the non-research related question of getting distracted by social media at work (2.19 (SD: 0.97) vs. 2.52 (1.19), *p* = 0.04) in comparison to non-active researchers and those without research experience. Further subgroup analyses are presented in the Supplemental Materials (b).

**Table 2:** Attitudes and opinions on a 5-point Likert scale: Work and personal life

	*N*	All	Active researchers	Others	*p*-value^a^	Survey questions^b^
Statement	All, active researchers/others	Mean (SD)	Mean (SD)	Mean (SD)		
Able to keep up with expertise	184, 82/102	3.73 (0.93)	3.95 (0.93)	3.55 (0.90)	0.003	27, 1
Feels bureaucracy at work is manageable	184, 82/102	3.22 (0.94)	3.15 (1.04)	3.28 (0.85)	0.34	27, 3
Feels like having control over work	184, 82/102	3.36 (1.04)	3.54 (1.02)	3.23 (1.04)	0.43	27, 4
Feels clinical workload is manageable	182, 80/102	3.48 (1.06)	3.66 (1.02)	3.34 (1.07)	0.41	27, 5
Feels on-call workload is manageable	154, 64/90	3.53 (1.13)	3.70 (1.15)	3.41 (1.11)	0.12	27, 6
Feels clinical software solutions are easy to learn	181, 80/101	3.44 (1.08)	3.53 (1.13)	3.37 (1.04)	0.33	27, 16
Feels the work environment is supportive	184, 82/102	4.33 (0.76)	4.34 (0.81)	4.32 (0.75)	0.88	27, 7
Positively affected by a mentor or a role model	178, 80/98	3.50 (1.09)	3.85 (0.97)	3.21 (1.11)	< 0.001	27, 17
Feels like having a good work-life balance	184, 82/102	3.06 (1.03)	3.49 (1.11)	3.58 (0.99)	0.56	27, 8
Gets distracted by social media at work	180, 80/100	2.37 (1.10)	2.19 (0.97)	2.52 (1.19)	0.04	27, 18
Feels they get enough sleep	183, 82/101	2.96 (1.11)	3.20 (1.09)	2.76 (1.09)	0.08	27, 19
Doesn’t have enough time for family	173, 78/95	2.97 (1.08)	2.88 (1.17)	3.03 (0.99)	0.38	28, 8
Considered leaving employment within the past year	178, 80/98	2.59 (1.32)	2.49 (1.70)	2.67 (1.34)	0.35	28, 3
Considered leaving medicine within the past year	182, 81/101	2.65 (1.25)	1.78 (1.11)	2.03 (1.22)	0.15	28, 1

*N* number, *SD* standard deviation

^a^ Statistical difference between active researchers and others

^b^ Indicates the question number of the Likert battery, followed by the number of the question in the battery. The survey is provided in Supplemental Materials

Average agreement with the following statements (1 = strongly disagree; 5 = strongly agree)

**Table 3:** Attitudes and opinions on a 5-point Likert scale: Research (1 = strongly disagree; 5 = strongly agree)

	*N*	All	Active researchers	Others	*p*-value^a^	Survey questions^b^
Statement	All, active researchers/others	Mean (SD)	Mean (SD)	Mean (SD)		
Wants to conduct medical research	180, 81/99	3.06 (1.40)	4.01 (1.10)	2.28 (1.11)	< 0.001	27, 9
Feels research is important for their career	179, 79/100	2.82 (1.27)	3.62 (1.07)	2.19 (1.04)	< 0.001	27, 11
Inspired by colleagues’ research work	181, 82/99	3.52 (1.11)	4.10 (0.80)	3.05 (1.11)	< 0.001	27, 12
Has the skills to do research	177, 82/95	3.34 (1.15)	3.96 (1.01)	2.80 (0.99)	< 0.001	27, 13
Feels training for research is available	145, 68/77	2.97 (1.03)	3.26 (1.02)	2.71 (0.99)	0.001	27, 14
Able to collaborate in research	154, 74/80	3.04 (1.22)	3.38 (1.21)	2.73 (1.15)	< 0.001	27, 15
Able to attain funding	143, 73/70	3.03 (1.00)	3.33 (0.88)	2.73 (1.03)	< 0.001	27, 10
Passed up on research due to lack of time	149, 77/72	3.25 (1.29)	3.45 (1.18)	3.03 (1.38)	0.045	28, 4
Passed up research because of family	162, 78/84	2.75 (1.29)	2.73 (1.26)	2.77 (1.32)	0.83	28, 7
Not suited for academic research	177, 81/96	2.59 (1.17)	2.11 (1.10)	3.00 (1.08)	< 0.001	28, 5
Considered quitting research (past year)	110, 78/32	2.59 (1.32)	2.58 (1.25)	2.84 (1.22)	0.31	28, 2
Does not get along with academic people	174, 81/93	1.84 (0.92)	1.69 (1.00)	1.97 (0.84)	0.051	28, 6
Interested in teaching	184, 82/102	3.82 (0.94)	4.27 (0.82)	3.45 (0.96)	0.001	27, 2

*N* number, *SD* standard deviation

^a^ Statistical difference between active researchers and others

^b^ Indicates the question number of the Likert battery followed by the number of the question in the battery. The survey is provided in the Supplemental Materials

The top self-identified choices for factors that may increase research involvement for respondents, regardless of previous research history, included dedicated time for research (94/155, 60.6%), motivation (56/155, 36.1%), more funding (36/155, 23.2%), and better salary (36/155, 23.2%).

We found a few significant differences in further subgroup analysis (complete tables available in the Supplemental Materials (b)).

The survey asked whether the respondents had children overall and if they had underage children living at home. All respondents who had children expressed higher agreement with the claim ‘I have passed up research opportunities because of family’ than those without children (2.08 (SD: 1.27) vs. 1.88 (SD: 0.96), *p* < 0.001). This was also true with those respondents with small children living at home (3.02 (SD: 1.23) vs. 2.45 (SD: 1.29) *p* = 0.004). Respondents with underage children living at home expressed higher agreement with the claims ’My colleagues and I support each other at work‘, (4.45 (SD: 0.68) vs. 4.21 (SD: 0.83), *p* = 0.05), ‘I don’t have enough time for my family’ (3.26 (SD: 1.03) vs. 2.63 (SD: 1.04), *p* < 0.001), and ‘I get distracted by social media at work’ (2.63 (SD: 1.15) vs. 2.09 (SD: 0.98), *p* < 0.001) than those without.

Relatively few significant differences were found between the genders. Male respondents expressed higher agreement with the claim ‘Overall I get enough sleep’ where the mean response for males was 3.23 (SD: 1.07), versus 2.73 (SD: 1.10) for females, with *p* = 0.003*.* Males also agreed slightly more with the claim ‘I feel that I have control over my work’ where the mean for males was 3.58 (SD: 0.98) vs. 3.17 (SD: 0.98) for females, *p* = 0.007. Female respondents expressed a slightly higher agreement with the claim ‘I get distracted by social media at work’ than males; Here the mean for female respondents was 2.52 (SD: 1.15), whereas it was 2.20 (SD: 1.03) for males, *p* = 0.07.

Finally, two differences between radiologists and residents indicated significance. Radiologists expressed higher agreement with having the necessary skills to do research compared to residents (3.43 (SD: 1.12) vs. 2.97 (SD: 1.21), *p* = 0.03). However, radiologists expressed lower agreement (3.36 (SD: 1.04), *p* = 0.002) with having a manageable clinical workload than residents 3.95 (SD: 1.01).

## Discussion

We investigated radiologist-dependent and external factors affecting the likelihood of conducting research among a cohort of Nordic radiologists. To study research involvement, we developed a survey and distributed it to radiologists and radiology residents of all career stages. According to our questionnaire, radiologists actively conducting research agreed more with several claims related to intrinsic and extrinsic motivators for research involvement in the questionnaire. They also reported having better access to the social and financial structures that support work in academic research. Surprisingly, we also found other positive tendencies, such as less distractibility by social media at work, the ability to maintain expertise, interest in teaching, and enough sleep. There were demonstrable differences in other subgroups as well. For instance, the experience of getting enough sleep was also expressed more by male respondents, while female respondents expressed more distractibility by social media and feeling less control over work. Radiologists expressed having a less manageable clinical workload than residents, but also higher agreement with having the skills to conduct research. Respondents with underage children at home expressed more distractibility by social media and were more likely to experience a supportive peer environment, as well as the desire for more time for family. Respondents with children agreed more with having experienced passing up research opportunities because of family.

Previous studies have reported that time spent on activities an individual deems meaningful can emphasise work immersion and lower the risk of physician burnout [32]. This might explain why our researchers are so motivated to perform their work, as well as to teach and learn. A review on postgraduate trainee research productivity found that [15] established research programmes, encouragement, and support could be factors that increase research productivity in residents. The same review, along with other studies, found mentorship to be helpful [16, 18]. Our study did not, by design, analyse programmatic aspects of the research environment, but we found supporting evidence on the positive effects of mentorship. Previous studies have reported that a gender gap favouring men in research activities may be narrowing [11, 13, 15, 16]. Other differences, such as distractibility by social media for some groups and sleep patterns between genders, are complex topics that warrant further study in the future to be better understood.

Self-identified factors that could increase research involvement highlighted time constraints, but these were not emphasised in the Likert questionnaire. This could be explained by active researchers having already overcome this hurdle in their careers at some level. Laupland et al found that protected time or scheduled blocks of research time enhance resident research productivity in most studies [15]. The self-identified need for motivation is either first or second on the list, depending on the group. This result is highly ambiguous, as our Likert results outlined the active researcher group to be already well motivated, and personal motivation is a subjective experience that can be affected by a myriad of both intrinsic and extrinsic factors. Therefore, the focus for future interventions targeting researcher workforce motivation should be tailored to the organisation in question as well as based on a current understanding of motivational theory. Both the Likert results and self-identified responses highlighted financial support as essential for research activity. Several studies have found significant increases in resident research productivity following monetary rewards [15]. Previous research also suggests that, beyond increased productivity, having a source of funding may increase research quality and the likelihood of citation [33]. Following these findings, the population of self-financed physicians conducting research on their own time may represent underutilised research potential. Overall, the responses form a hierarchy that can help with planning possible interventions targeting researcher workforce productivity, with necessary scrutiny due to the subjective nature of the responses.

There are several limitations to consider in regard to the findings presented here. Firstly, the study had a limited cohort size despite active recruitment. Secondly, the factors studied here may be affected by recent and concurrent world events such as conflicts, the COVID-19 pandemic, and economic cycles. We plan to control for these external factors by repeating the survey after two years in 2025 for comparison. Thirdly, the results stem from a Nordic cohort of volunteers, which may result in selection bias. To what extent these results may be generalised to radiologists in other countries is unclear. Similarly, differences in health policies and cultural and organisational differences may affect the generalisability of the results.

In conclusion, we found that allocated time, as well as better access to social and financial structures supporting research, have a positive association with research involvement. Research involvement was also associated with better sleep, less distractibility by social media, and being able to maintain expertise and interest in teaching. Further research is necessary to establish the generalisability of these results in other medical specialties and populations outside the surveyed Nordic countries.

## Supplementary information

ELECTRONIC SUPPLEMENTARY MATERIAL

## Data Availability

Original data is contained in the Pirha RedCap platform and the secure Lokero database. Datasets containing personally identifiable information may not be produced to third parties as stated in the GDPR-compliant study data policy. As per the study data management plan, data may not be provided to third parties as a combination of personal and professional details might result in the anonymity of respondents being compromised.

## Abbreviations

COVID-19: Coronavirus disease 2019
REDCap: Research Electronic Data Capture platform

## References

[1] Ghasemi A, Mirmiran P, Kashfi K, Bahadoran Z (2023) Scientific publishing in biomedicine: a brief history of scientific journals. Int J Endocrinol Metab 21:1–10. 10.5812/ijem-131812 10.5812/ijem-131812 PMC10024814 36945344

[2] Zhang LJ, Wang YF, Yang ZL et al (2017) Radiology research in mainland China in the past 10 years: a survey of original articles published in Radiology and European Radiology. Eur Radiol 27:4379–4382. 10.1007/s00330-016-4689-4 28332016 10.1007/s00330-016-4689-4

[3] Ho ML, Arnold CW, Decker SJ, Hazle JD, Krupinski EA, Mankoff DA (2023) Institutional strategies to maintain and grow imaging research during the COVID-19 pandemic. Acad Radiol 30:631–639. 10.1016/j.acra.2022.12.045 10.1016/j.acra.2022.12.045 PMC9816088 36764883

[4] Vagal A, Reeder SB, Sodickson DK, Goh V, Bhujwalla ZM, Krupinski EA (2020) The impact of the COVID-19 pandemic on the radiology research enterprise: radiology scientific expert panel. Radiology 296:E134–E140. 10.1148/radiol.2020201393 32293224 10.1148/radiol.2020201393 PMC7233405

[5] Dandena A, Kebede T (2022) Attitude, practice and barriers of academic research among radiology residents in Ethiopia: a cross-sectional survey. Ethiop J Health Sci 32:61–68. 10.4314/ejhs.v32i1.10S 36339961 10.4314/ejhs.v32i1.10S PMC9624097

[6] Hames K, Patlas M, Duszak R (2018) Barriers to resident research in radiology: a Canadian perspective. Can Assoc Radiologist J 69:260–265. 10.1016/j.carj.2018.03.006 10.1016/j.carj.2018.03.006 30078398

[7] McGrail MR, O’Sullivan BG, Bendotti HR, Kondalsamy-Chennakesavan S (2019) Importance of publishing research varies by doctors’ career stage, specialty and location of work. Postgrad Med J 95:198–204. 10.1136/postgradmedj-2019-136473 30926718 10.1136/postgradmedj-2019-136473

[8] Lopes J, Ranieri V, Lambert T et al (2017) The clinical academic workforce of the future: a cross-sectional study of factors influencing career decision-making among clinical PhD students at two research-intensive UK universities. BMJ Open 7:1–19. 10.1136/bmjopen-2017-016823 10.1136/bmjopen-2017-016823 PMC5724086 28851792

[9] Rahal A, Head HW, Jung AJ et al (2007) Combined radiology residency/PhD program for education of academic radiologists: a response to revitalizing the radiology research enterprise. Radiology 245:14–20. 10.1148/radiol.2451070168 17885177 10.1148/radiol.2451070168 PMC3675225

[10] Pretorius ES, Solomon JA, Stribling C (2003) Medical student attitudes toward inclusion of a research year within diagnostic radiology residency: a survey of students participating in the 2002 NRMP match. Acad Radiol 10:77–82. 10.1016/S1076-6332(03)80792-4 12529033 10.1016/s1076-6332(03)80792-4

[11] Jagsi R, Guancial EA, Worobey CC et al (2006) The “gender gap” in authorship of academic medical literature—a 35-year perspective. N Engl J Med. 281–287. 10.1056/NEJMsa053910. 10.1056/NEJMsa053910 16855268

[12] Oliveira DFM, Ma Y, Woodruff TK, Uzzi B (2019) Comparison of National Institutes of Health Grant amounts to first-time male and female principal investigators. J Am Med Assoc 321:898–900. 10.1001/jama.2018.21944 10.1001/jama.2018.21944 PMC6439593 30835300

[13] Komlenac N, Gustafsson Sendén M, Verdonk P, Hochleitner M, Siller H (2019) Parenthood does not explain the gender difference in clinical position in academic medicine among Swedish, Dutch and Austrian physicians. Adv Health Sci Educ 24:539–557. 10.1007/s10459-019-09882-9 10.1007/s10459-019-09882-9 PMC6647470 30840215

[14] O’Neill SB, Maddu K, Jalal S et al (2019) Gender disparity in chest radiology in North America. Curr Probl Diagn Radiol 000:1–5. 10.1067/j.cpradiol.2019.10.001 10.1067/j.cpradiol.2019.10.001 31732263

[15] Laupland KB, Edwards F, Dhanani J (2021) Determinants of research productivity during postgraduate medical education: a structured review. BMC Med Educ 21:1–9. 10.1186/s12909-021-03010-1 34753470 10.1186/s12909-021-03010-1 PMC8579624

[16] Borges NJ, Navarro AM, Grover A, Hoban JD (2010) How, when, and why do physicians choose careers in academic medicine? A literature review. Acad Med 85:680–686. 10.1097/ACM.0b013e3181d29cb9 20354389 10.1097/ACM.0b013e3181d29cb9

[17] Decker SJ, Grajo JR, Hazelton TR et al (2016) Research challenges and opportunities for clinically oriented academic radiology departments. Acad Radiol 23:43–52. 10.1016/j.acra.2015.10.007 26598485 10.1016/j.acra.2015.10.007

[18] Krupinski EA, Votaw JR (2015) Research resources survey: radiology junior faculty development. Acad Radiol 22:918–932. 10.1016/j.acra.2015.02.010 26251861 10.1016/j.acra.2015.02.010

[19] Marjavaara S, Illman S, Rämet M (2017) Kliinisen tutkijan kysely. https://www.aka.fi/globalassets/2-suomen-akatemian-toiminta/4-julkaisut/julkaisut/kliinisen-tutkijan-kysely-2017.pdf. Accessed 27 Jul 2025

[20] Nuutinen TA, Mälkki A (2016) Tieteen tila. https://www.aka.fi/globalassets/2-suomen-akatemian-toiminta/2-tietoaineistot/aka_tieteen_tila_yksi.pdf. Accessed 27 Jul 2025

[21] Aula Research Oy (2019) Lääketieteen tutkimusrahoitus 2010-luvulla. https://www.yjs.fi/wp-content/uploads/selvitys-laaketieteen-tutkimusrahoituksesta.pdf. Accessed 7 Oct 2025

[22] Shankar PR, Maturen KE (2019) Survey research reporting in radiology publications: a review of 2017 to 2018. J Am Coll Radiol 16:1378–1384. 10.1016/j.jacr.2019.07.012 31585659 10.1016/j.jacr.2019.07.012

[23] Rotenstein LS, Torre M, Ramos MA et al (2018) Prevalence of burnout among physicians a systematic review. J Am Med Assoc 1131–1150. 10.1001/jama.2018.12777 10.1001/jama.2018.12777 PMC6233645 30326495

[24] Revilla M, Höhne JK (2020) How long do respondents think online surveys should be? New evidence from two online panels in Germany. Int J Mark Res 62:538–545. 10.1177/1470785320943049

[25] Den Norske Legeforening (2025) Legestatistikk. https://www.legeforeningen.no/om-oss/legestatistikk/. Accessed 27 Jul 2025

[26] Terveyden ja Hyvinvoinnin Laitos (2025) Social and healthcare professional rights and professional rights holders in Finland. https://sampo.thl.fi/pivot/prod/fi/henkilo/valvira/fact_henkilo_valvira?row=occup-1183030&column=time-566596.566528.566650.566604.566612.629424.701905.980572.1118605.1183075.&filter=age-1183084. Accessed 27 Jul 2025

[27] Dansk Radiologisk Selskab (2025) om Radiologisk Selskab. https://www.drs.dk/radiologiskselskab/. Accessed 27 Jul 2025

[28] Brady AP, Paulo G, Brkljacic B, Loewe C, Szucsich M, Hierath M (2025) Current status of radiologist staffing, education and training in the 27 EU Member States. Insights Imaging. 16. 10.1186/s13244-025-01925-7 10.1186/s13244-025-01925-7 PMC11910488 40088348

[29] Van Mol C (2017) Improving web survey efficiency: the impact of an extra reminder and reminder content on web survey response. Int J Soc Res Methodol 20:317–327. 10.1080/13645579.2016.1185255

[30] Phillips AW, Reddy S, Durning SJ (2016) Improving response rates and evaluating nonresponse bias in surveys: AMEE Guide No. 102. Med Teach. Taylor and Francis Ltd. pp. 217–228. 10.3109/0142159X.2015.1105945. 10.3109/0142159X.2015.1105945 26648511

[31] Norman G (2010) Likert scales, levels of measurement and the “laws” of statistics. Adv Health Sci Educ 15:625–632. 10.1007/s10459-010-9222-y 10.1007/s10459-010-9222-y 20146096

[32] Shanafelt TD, West CP, Sloan JA et al (2009) Career fit and burnout among academic faculty. Arch Intern Med 169:990–995. 10.1001/archinternmed.2009.70 19468093 10.1001/archinternmed.2009.70

[33] Ebadi A, Schiffauerova A (2016) How to boost scientific production? A statistical analysis of research funding and other influencing factors. Scientometrics 106:1093–1116. 10.1007/s11192-015-1825-x

