# Supplementary material for: Factors associated with research activity among radiologists: results of a Nordic survey
Source: Insights Imaging. 2025 Oct 26;16:230. doi: 10.1186/s13244-025-02108-0 (PMC12554850; doi:10.1186/s13244-025-02108-0)

Supplemental material for:

”The Nordic survey assessing radiologists' motivators and stressors”

Survey questions numbered and written out for reference:

In this version of the questionnaire, questionnaire sections and questions have been numbered and questions, as well as response choices have been labelled for convenient reference.

Survey data policy

Please read and accept the privacy policy, if you wish to continue.

1. ***What we collect and how***
   We will collect personal data such as your age, gender and e-mail address. We will also collect data regarding your personal experiences, opinions and achievements. Your e-mail address is collected to confirm responders as individuals, to inform you of the results and to send a follow-up survey in approximately two years to establish trends. At no circumstance will we provide your contact information to third parties. All data will be collected through the survey. IP-address, geolocation or similar metadata will not be collected by the research team.

2. ***Why we collect***
   We collect data for the purpose of scientific research. Personal identifying data such as contact information will only be used for the purpose of confirming responses as individual responses, to inform you of the study results and to send a follow-up survey in two years.

3. ***How we will you use your data***
   We will only use your data for the purposes of scientific research, and for contacting you in the case that it is directly necessitated by the research procedure or law.

4. ***How long we will keep your data***
   Data regarding personal identity or contact information will be deleted after it is no longer necessary for this research initiative, or after a maximum of five years after the end of this research study (by the end of January 2034).

5. ***How your data will be secured***
   We will only store and handle your personal data in the General Data Protection Regulation (GDPR) compliant hospital network.

6. ***Respondent rights***
   You can end the survey at any time, the data collected until this point can be used for research purposes. As we do not verify your identity, you do not have the right to access your responses after submitting them. You have the right to request us to delete the information you have provided by contacting ____________ during the time of this survey, and until all the research data is deleted. All your retained personal data will be permanently deleted after this, but it will not be possible to remove or change your response data from anonymized datasets or derived analyses created before the request.

   You have a right to opt out of any targeted follow-up communication. Please note that you may still receive non-targeted communication related to the results of this survey and an invitation to participate to a follow-up survey in approximately two years.

7. ***Whom to contact***
   The registrar of all data collected by this initiative is the ____________. All questions regarding the survey, related research or data collection, processing or storage should be directed to the initiative's data protection officer ____________.

Response choices:

- ○ I have read the terms and conditions of this study and consent to my data being used anonymously for the purposes of scientific research. I understand that the legal basis for processing my data is public interest.
- ○ I do not accept the terms and conditions of this study, and will not continue.

Section 1: Personal information

Question 1:

Please enter your valid e-mail address.

Response:

[Text box]

Question 2:

Which of the following describes you best:

Response choices:

- Radiologist
- Resident
- Other, please specify [Text box]

Question 3:

How old are you in years?

Response choices:

[One choice multiple choice field, response options between 16 and 100]

Question 4:

What is your gender?

Response choices:

- Male
- Female
- Other
- Prefer not to say

Question 5:

What is your country of residence?

Response choices:

- Denmark
- Finland
- Norway
- Sweden
- Other

Question 6:

Do you have children?

Response choices:

- Yes
- No

Question 7:

How many underage children (under the age of 18) do you have living at home?

Response:

[Text box]

Question 8:

Which best describes your civil status?

Response choices:

- Married
- In a civil union recognized by law or equivalent
- In a committed relationship
- Single
- Widowed
- Divorced
- I prefer not to say
- Other

Section 2: Information on work and research background

Question 9:

What is your clinical work status?

Response choices:

- Working full time
- Working part time
- Not doing clinical work

Question 10:

What best describes your current research work status?

Response choices:

- Doing full time research
- Doing part time research
- Doing research mainly on free time
- Not doing research

Question 11:

What best describes your employment status?

Response choices:

- One employer
- Several employers

- Unemployed

Question 12:

What is your primary work sector (if applicable)?

Response choices:

- Public sector
- Government (local or national)
- Military
- Private sector
- Charity, trust or other non-governmental organisation
- Other

Question 13:

How many years of experience do you have in the field of radiology?

Response:

[Free text box]

Question 14:

Please evaluate your proficiency in the following fields

Response choices:

- Breast radiology
- Cardiac and vascular radiology

- Emergency radiology
- Gastrointestinal and abdominal radiology
- Gynaecological and obstetric radiology
- Head and neck radiology
- Interventional radiology
- Musculoskeletal radiology
- Neuroradiology
- Oncologic imaging
- Paediatric radiology
- Urogenital radiology
- Nuclear medicine

Question 15:

Have you ever contributed to an academic research project (e.g. by co-authoring a research paper or otherwise contributing to a research project)?

Response choices:

- Yes
- No

Question 16:

Are you participating, or have you participated in research training after a PhD, such as a post doctoral

Response choices:

- Finished a post doc
- Participating in a post doc program?

- Planning on doing a post doc
- No
- Other [Text box]

Question 17:

How many peer-reviewed publications have you authored, if any?

Response:

[Text box]

Question 18:

How many publications do you currently have in review, if any?

Response:

[Text box]

Question 19:

How many years of research experience do you have?

Response:

[Text box]

Question 20:

Please describe your research experience

Response choices:

- ☐ Consulted a research group without direct participation
- ☐ Worked as a member of a research group
- ☐ Worked as a supervisor of a research group
- ☐ Other [Text box]

Question 21:

What different types of research studies have you conducted? Please check all that apply.

Response choices:

- ☐ Imaging methods or technology
- ☐ Clinical research
- ☐ Register based study
- ☐ Randomised controlled trial (RCT)
- ☐ Prospective
- ☐ Retrospective
- ☐ Cohort study
- ☐ Cross-sectional
- ☐ Interventional
- ☐ Systematic review
- ☐ Meta-analysis
- ☐ Case report
- ☐ Case series
- ☐ Editorial, opinion
- ☐ Animal research study
- ☐ Other [Text box]

Question 22:

Check all that apply. Who has funded your research in the past year?

Response choices:

- ☐ Public sector employer
- ☐ Private sector employer
- ☐ Charity
- ☐ Government
- ☐ Military
- ☐ University
- ☐ Pharmaceutical industry
- ☐ Medical technology industry
- ☐ Out of pocket/Self-funded
- ☐ Other [Text box]

Question 23:

Check all that apply. Who has funded your research in the past five years?

Response choices:

- ☐ Public sector employer
- ☐ Private sector employer
- ☐ Charity
- ☐ Government
- ☐ Military
- ☐ University
- ☐ Pharmaceutical industry

- ☐ Medical technology industry
- ☐ Out of pocket/Self-funded
- ☐ Other [Text box]

Question 24:

Check all degrees or academic titles that currently apply to you

Response choices:

- ☐ Professor
- ☐ Associate professor or equivalent
- ☐ PhD
- ☐ MD
- ☐ Undergraduate/medical student (MBBS)
- ☐ Other [Text box]

Section 3: Work engagement and burnout

Question 25:

Ultra-short work engagement survey (UWES)

Questions:

1. At my work, I feel bursting with energy
2. I am enthusiastic about my job
3. I am immersed in my work

Response choices:

1. Never
2. A few times a year
3. Once a month
4. A few times a month
5. Once a week
6. A few times a week
7. Every day

Question 26:

Burnout assessment tool (BAT)

Questions:

At work, I feel mentally exhausted

1. I struggle to find any enthusiasm for my work

2. At work, I feel unable to control my emotions
3. When I'm working, I have trouble concentrating

Response choices:

1. Never
2. Rarely
3. Sometimes
4. Often
5. Always

Question 27:

Answer how much you disagree or agree with the following statements.

Questions:

1. I can study to keep my expertise up to date
2. I am interested in teaching
3. The bureucracy at work is manageable
4. I feel that I have control over my work
5. My clinical workload is manageable
6. My on-call duty load is manageable
7. My colleagues and I support each other at work
8. I have a good balance between work and personal life
9. I want to conduct medical research
10. I could get funding for research if I needed it
11. Research is important for my career plans
12. I am inspired by a colleague or colleagues who conduct research
13. I have the necessary skills to do research
14. Research-oriented programs or training are available for me

15. I could join a multi-institution or international research collaboration at my primary place of employment
16. The clinical software solutions I use at work are easy for me to learn
17. My career has been positively affected by a mentor or a role model
18. I get distracted by social media at work
19. Overall, I get enough sleep

Response choices on a 5 point likert scale:

1. Strongly disagree
2. Disagree
3. Neutral
4. Agree
5. Strongly agree
6. Don't know/not applicable

Section 4: Personal views and experiences

Question 28 as a mini-questionnaire:

Questions:

1. Within the past year I have considered leaving medicine
2. Within the past year I have considered quitting research (if applicable)
3. Within the past year I have considered leaving my current employment
4. I have passed up research opportunities because of time constraints
5. I don't think I am suited for academic research
6. I don't get along with academic people
7. I have passed up research opportunities because of my family
8. I don't have enough time for my family

Response choices on a 5 point likert scale:

1. Strongly disagree
2. Disagree
3. Neutral
4. Agree
5. Strongly agree
6. Don't know/not applicable

Question 29:

What would make you get more involved with research

(choose up to 3 answers)

Response choices:

- Motivation to do so
- Funding
- Time for research at work
- Support from my employer
- Fewer clinical duties
- Prestige or awards
- Career advancement opportunities
- Better salary
- More researchers in my personal network
- A role model
- A mentor
- Research training
- Nothing would get me involved in research
- Other [Text box]

Question 30:

Have you been discriminated against in the academic environment? If you wish, you may elaborate in the "other" field.

Response choices:

- ☐ No
- ☐ Because of gender
- ☐ Because of sexuality
- ☐ Because of race
- ☐ Because of religion
- ☐ Because of political opinions
- ☐ Because of union activity
- ☐ Other [Text box]

Section 5: Charity giveaway and feedback

Thank you for responding to our survey. If you have any feedback, you can enter it in the box below.

Response:

[Text box]

Do you want to be informed of this study's publication?

Response choices:

- Yes
- No

Do you wish to receive a personal reminder for the follow-up survey in two years?

Response choices:

- Yes
- No

We are giving away a sum of 100€ to a charity for each surveyed country that exceeds 300 responses. You may vote for a charity below

Response choices:

- International Red Cross (icrc.org/en)
- Doctors Without Borders (www.msf.org)
- Save the Children (savethechildren.net)
- World Vision International (wvi.org)
- World Food Programme (wfp.org)

- No answer

Thank you kindly for your responses.

We need your help! After you complete your responses, please forward the survey link to other radiologists through your professional networks.

Supplemental tables and figures for:
"Factors Associated with Research Activity Among Radiologists: Results from A Nordic Survey"

Tables including result comparisons between:

Men and women

Radiologists and residents

Radiologists with and without children

Radiologists with and without underage children living at home

Insights Imaging (2025) Jylhä-Vuorio P, Rinta-Kiikka I, Mäkikangas A, Hirvonen J, Luukkaala T, Arponen O

Supplemental Table 1: Attitudes and opinions on work and personal life on a 5-point Likert scale. Average agreement with the following statements (1=strongly disagree; 5=strongly agree).

**Men and Women**	N	All	Male	Female	*P*-value [a]	Survey question [b]
Statement	All, male / female	Mean (SD)	Mean (SD)	Mean (SD)		
Able to keep up expertise	183, 84/99	3.73 (0.93)	3.82 (0.89)	3.65 (0.96)	.20	27, 1
Feels bureaucracy at work is manageable	183, 84/99	3.22 (0.94)	3.31 (1.02)	3.15 (0.87)	.22	27, 3
Feels having control over work	183, 84/99	3.36 (1.04)	3.58 (0.98)	3.17 (0.98)	.007	27, 4
Feels clinical workload is manageable	181, 83/98	3.48 (1.06)	3.53 (1.12)	3.44 (1.01)	.43	27, 5
Feels on-call workload is manageable	153, 67/86	3.53 (1.13)	3.66 (1.12)	3.43 (1.14)	.19	27, 6
Feels clinical software solutions are easy to learn	180, 82/98	3.44 (1.08)	3.60 (1.08)	3.30 (1.07)	.81	27, 16
Feels work environment is supportive	183, 84/99	4.33 (0.76)	4.42 (0.75)	4.25 (0.77)	.10	27, 7
Positively affected by a mentor or a role model	177, 81/96	3.50 (1.09)	3.59 (1.14)	3.44 (1.04)	.30	27, 17
Feels having a good work-life balance	183, 84/99	3.06 (1.03)	3.57 (1.06)	3.51 (1.02)	.66	27, 8

Gets distracted by social media at work	179, 81/98	2.37 (1.10)	2.20 (1.03)	2.52 (1.15)	.07	27, 18
Feels they get enough sleep	182, 83/99	2.96 (1.11)	3.23 (1.07)	2.73 (1.10)	.003	27, 19
Doesn't have enough time for family	172, 80/92	2.97 (1.08)	2.89 (1.08)	3.04 (1.08)	.42	28, 8
Considered leaving employment within past year	177, 82/95	2.59 (1.32)	2.54 (1.34)	2.63 (1.32)	.62	28, 3
Within the past year I have considered leaving medicine	181, 83/98	2.65 (1.25)	1.78 (1.07)	2.04 (1.25)	.24	28, 1

Abbreviations: N=number; SD=standard deviation

[a] Statistical difference between men and women

[b] Indicates the question number of the Likert battery followed by the number of the question in the battery. The survey is provided in Supplemental materials.

Supplemental Table 2: Attitudes and opinions on a 5-point Likert scale: Research. Average agreement with the following statements (1=strongly disagree; 5=strongly agree).

**Men and Women**	N	All	Male	Female	*P*-value [a]	Survey question [b]
Statement	All, male / female	Mean (SD)	Mean (SD)	Mean (SD)		
Wants to conduct medical research	179, 83/96	3.06 (1.40)	3.02 (1.49)	3.09 (1.33)	.79	27, 9
Feels research is important for career	178, 82/96	2.82 (1.27)	2.74 (1.27)	2.89 (1.28)	.45	27, 11
Inspired by colleagues' research work	180, 82/98	3.52 (1.11)	3.48 (1.21)	3.57 (1.03)	.72	27, 12
Has skills to do research	176, 81/95	3.34 (1.15)	3.36 (1.14)	3.15 (1.16)	.75	27, 13
Feels training for research is available	144, 67/77	2.97 (1.03)	3.15 (1.08)	2.82 (0.98)	.051	27, 14
Able to collaborate in research	153, 71/82	3.04 (1.22)	3.03 (1.25)	3.02 (1.19)	.99	27, 15
Able to attain funding	142, 68/74	3.03 (1.00)	3.18 (0.98)	2.89 (1.02)	.055	27, 10
Passed up research due to lack of time	148, 71/77	3.25 (1.29)	3.06 (1.38)	3.42 (1.20)	.11	28, 4
Passed up research because of family	161, 75/86	2.75 (1.29)	2.63 (1.24)	2.84 (1.31)	.30	28, 7
Not suited for academic research	176, 82/94	2.59 (1.17)	2.49 (1.14)	2.69 (1.20)	.30	28, 5

Considered quitting research (past year)	109, 50/59	2.59 (1.32)	2.70 (1.40)	2.59 (1.10)	.77	28, 2
Does not get along with academic people	173, 81/92	1.84 (0.92)	1.84 (0.98)	1.85 (0.88)	.72	28, 6
Interested in teaching	183, 84/99	3.82 (0.94)	3.89 (0.93)	3.76 (1.03)	.51	27, 2

Abbreviations: N=number; SD=standard deviation

[a] Statistical difference between men and women

[b] Indicates the question number of the Likert battery followed by the number of the question in the battery. The survey is provided in Supplemental materials.

Supplemental Table 3: Attitudes and opinions on a 5-point Likert scale: Work, and personal life. Average agreement with the following statements (1=strongly disagree; 5=strongly agree).

**Radiologists and residents**	N	All	Radiologist	Resident	*P*-value [a]	Survey question [b]
Statement	All, radiologist / resident	Mean (SD)	Mean (SD)	Mean (SD)		
Able to keep up expertise	184, 146/38	3.73 (0.93)	3.69 (0.95)	3.87 (0.84)	.33	27, 1
Feels bureaucracy at work is manageable	184, 146/38	3.22 (0.94)	3.17 (0.90)	3.42 (1.08)	.11	27, 3
Feels having control over work	184, 146/38	3.36 (1.04)	3.35 (1.00)	3.42 (1.20)	.48	27, 4
Feels clinical workload is manageable	182, 144/38	3.48 (1.06)	3.36 (1.04)	3.95 (1.01)	.002	27, 5
Feels on-call workload is manageable	154, 119/35	3.53 (1.13)	3.63 (1.04)	3.20 (1.37)	.10	27, 6
Feels clinical software solutions are easy to learn	181, 145/36	3.44 (1.08)	3.37 (1.07)	3.69 (1.06)	.06	27, 16
Feels work environment is supportive	184, 146/38	4.33 (0.76)	4.31 (0.75)	4.42 (0.83)	.24	27, 7

Positively affected by a mentor or a role model	178, 141/37	3.50 (1.09)	3.47 (1.10)	3.62 (1.06)	.32	27, 17
Feels having a good work-life balance	184, 146/38	3.06 (1.03)	3.49 (0.99)	3.71 (1.18)	.19	27, 8
Gets distracted by social media at work	180, 143/37	2.37 (1.10)	2.35 (1.12)	2.46 (1.07)	.54	27, 18
Feels they get enough sleep	183, 146/37	2.96 (1.11)	2.96 (1.13)	2.95 (1.05)	.97	27, 19
Doesn’t have enough time for family	173, 139/34	2.97 (1.08)	2.94 (1.09)	3.06 (1.04)	.62	28, 8
Considered leaving employment within past year	178, 143/35	2.59 (1.32)	2.73 (1.31)	2.03 (1.25)	.003	28, 3
Considered leaving medicine within past year	182, 145/37	2.65 (1.25)	1.92 (1.15)	1.92 (1.30)	.66	28, 1

Abbreviations: N=number; SD=standard deviation

[a] Statistical difference between radiologists and residents

[b] Indicates the question number of the Likert battery followed by the number of the question in the battery. The survey is provided in Supplemental materials.

Supplemental Table 4: Attitudes and opinions on a 5-point Likert scale: Research (1=strongly disagree; 5=strongly agree).

**Radiologists and residents**	N	All	Radiologist	Resident	*P*-value [a]	Survey question [b]
Statement		Mean (SD)	Mean (SD)	Mean (SD)		
Wants to conduct medical research	180, 143/37	3.06 (1.40)	3.05 (1.41)	3.11 (1.37)	.82	27, 9
Feels research is important for career	179, 143/36	2.82 (1.27)	2.78 (1.25)	3.00 (1.77)	.37	27, 11
Inspired by colleagues’ research work	181, 144/37	3.52 (1.11)	3.45 (1.12)	3.81 (1.05)	.06	27, 12
Has skills to do research	177, 141/36	3.34 (1.15)	3.43 (1.12)	2.97 (1.21)	.03	27, 13
Feels training for research is available	145, 119/26	2.97 (1.03)	2.99 (1.01)	2.88 (1.14)	.69	27, 14
Able to collaborate in research	154, 129/25	3.04 (1.22)	3.06 (1.24)	2.92 (1.12)	.60	27, 15

Able to attain funding	143, 114/29	3.03 (1.00)	3.02 (1.03)	3.10 (0.90)	.69	27, 10
Passed up research due to lack of time	149, 119/30	3.25 (1.29)	3.29 (1.27)	3.10 (1.40)	.56	28, 4
Passed up research because of family	162, 133/29	2.75 (1.29)	2.77 (1.26)	2.69 (1.44)	.70	28, 7
Not suited for academic research	177, 142/35	2.59 (1.17)	2.51 (1.14)	2.91 (1.25)	.08	28, 5
Considered quitting research (past year)	110, 92/18	2.59 (1.32)	2.66 (1.23)	2.61 (1.34)	.88	28, 2
Does not get along with academic people	174, 141/33	1.84 (0.92)	1.86 (0.91)	1.76 (1.00)	.36	28, 6
Interested in teaching	184, 146/38	3.82 (0.94)	3.79 (1.01)	3.92 (0.88)	.62	27, 2

Abbreviations: N=number; SD=standard deviation

[a] Statistical difference between radiologists and residents

[b] Indicates the question number of the Likert battery followed by the number of the question in the battery. The survey is provided in Supplemental materials.

Supplemental Table 5: Attitudes and opinions on a 5-point Likert scale. Work, and personal life. Average agreement with the following statements (1=strongly disagree; 5=strongly agree).

**Radiologists with and without children**	N	All	Has children	Has no children	*P*-value [a]	Survey question [b]
Statement	All, has children / has no children	Mean (SD)	Mean (SD)	Mean (SD)		
Able to keep up expertise	184, 141/43	3.73 (0.93)	3.72 (0.94)	3.77 (0.90)	.85	27, 1
Feels bureaucracy at work is manageable	184, 141/43	3.22 (0.94)	3.21 (0.89)	3.28 (1.10)	.66	27, 3
Feels having control over work	184, 141/43	3.36 (1.04)	3.38 (1.03)	3.33 (1.09)	.79	27, 4
Feels clinical workload is manageable	182, 139/43	3.48 (1.06)	3.47 (1.04)	3.51 (1.12)	.82	27, 5

Feels on-call workload is manageable	154, 117/37	3.53 (1.13)	3.56 (1.13)	3.46 (1.17)	.67	27, 6
Feels clinical software solutions are easy to learn	181, 139/42	3.44 (1.08)	3.44 (1.05)	3.43 (1.17)	.93	27, 16
Feels work environment is supportive	184, 141/43	4.33 (0.76)	4.38 (0.69)	4.16 (0.95)	.29	27, 7
Positively affected by a mentor or a role model	178, 137/41	3.50 (1.09)	3.58 (1.08)	3.22 (1.11)	.07	27, 17
Feels having a good work-life balance	184, 141/43	3.06 (1.03)	3.53 (1.04)	3.56 (1.03)	.85	27, 8
Gets distracted by social media at work	180, 138/42	2.37 (1.10)	2.45 (1.10)	2.12 (1.09)	.07	27, 18
Feels they get enough sleep	183, 140/43	2.96 (1.11)	2.96 (1.13)	2.93 (1.06)	.86	27, 19
Doesn't have enough time for family	173, 140/33	2.97 (1.08)	3.03 (1.09)	2.70 (0.98)	.10	28, 8
Considered leaving employment within past year	178, 138/40	2.59 (1.32)	2.60 (1.30)	2.55 (1.41)	.76	28, 3
Considered leaving medicine within past year	182, 140/42	2.65 (1.25)	1.84 (1.11)	2.17 (1.34)	.21	28, 1

Abbreviations: N=number; SD=standard deviation

[a] Statistical difference between those with children and those without

[b] Indicates the question number of the Likert battery followed by the number of the question in the battery. The survey is provided in Supplemental materials.

Supplemental Table 6: Attitudes and opinions on a 5-point Likert scale: Research. Average agreement with the following (1=strongly disagree; 5=strongly agree).

**Radiologists with and without children**	N	All	Has children	Has no children	*P*-value [a]	Survey question [b]
Statement	All, has children / has no children	Mean (SD)	Mean (SD)	Mean (SD)		
Wants to conduct medical research	180, 138/42	3.06 (1.40)	3.10 (1.37)	2.93 (1.49)	.50	27, 9
Feels research is important for career	179, 137/42	2.82 (1.27)	2.80 (1.20)	2.90 (1.48)	.76	27, 11
Inspired by colleagues' research work	181, 138/43	3.52 (1.11)	3.60 (1.06)	3.28 (1.24)	.11	27, 12
Has skills to do research	177, 134/43	3.34 (1.15)	3.44 (1.09)	3.02 (1.30)	.07	27, 13

Feels training for research is available	145, 114/31	2.97 (1.03)	2.98 (1.03)	2.94 (1.06)	.71	27, 14
Able to collaborate in research	154, 125/29	3.04 (1.22)	3.10 (1.23)	2.76 (1.15)	.15	27, 15
Able to attain funding	143, 112/31	3.03 (1.00)	3.14 (1.01)	3.00 (1.00)	.82	27, 10
Passed up research due to lack of time	149, 115/34	3.25 (1.29)	3.34 (1.28)	2.94 (1.30)	.11	28, 4
Passed up research because of family	162, 129/33	2.75 (1.29)	2.98 (1.27)	1.88 (0.96)	<.001	28, 7
Not suited for academic research	177, 136/41	2.59 (1.17)	2.50 (1.12)	2.90 (1.28)	.11	28, 5
Considered quitting research (past year)	110, 89/21	2.59 (1.32)	2.56 (1.17)	3.05 (1.50)	.17	28, 2
Does not get along with academic people	174, 135/39	1.84 (0.92)	1.82 (0.95)	1.90 (0.85)	.38	28, 6
Interested in teaching	184, 141/43	3.82 (0.94)	3.84 (0.92)	3.74 (1.18)	.90	27, 2

Abbreviations: N=number; SD=standard deviation

[a] Statistical difference between those with children and those without

[b] Indicates the question number of the Likert battery followed by the number of the question in the battery. The survey is provided in Supplemental materials.

Supplemental table 7: Attitudes and opinions on a 5-point Likert scale: Work, and personal life. Average agreement with the following statements (1=strongly disagree; 5=strongly agree).

**Radiologists with and without underage children living at home**	N	All	Has UC	Has no UC	*P*-value [a]	Survey question [b]
Statement	All, has UC / has no UC	Mean (SD)	Mean (SD)	Mean (SD)		
Able to keep up expertise	184, 94/90	3.73 (0.93)	3.62 (1.00)	3.84 (0.85)	.14	27, 1
Feels bureaucracy at work is manageable	184, 94/90	3.22 (0.94)	3.23 (0.92)	3.21 (0.97)	.79	27, 3
Feels having control over work	184, 94/90	3.36 (1.04)	3.27 (1.07)	3.47 (1.01)	.24	27, 4
Feels clinical workload is manageable	182, 94/88	3.48 (1.06)	3.53 (1.07)	3.43 (1.05)	.47	27, 5
Feels on-call workload is manageable	154, 85/69	3.53 (1.13)	3.62 (1.13)	3.42 (1.13)	.25	27, 6

Feels clinical software solutions are easy to learn	181, 93/88	3.44 (1.08)	3.43 (1.08)	3.21 (1.08)	.88	27, 16
Feels work environment is supportive	184, 94/90	4.33 (0.76)	4.45 (0.68)	4.21 (0.83)	.05	27, 7
Positively affected by a mentor or a role model	178, 91/87	3.50 (1.09)	3.65 (1.06)	3.34 (1.11)	.07	27, 17
Feels having a good work-life balance	184, 94/90	3.06 (1.03)	3.43 (1.03)	3.66 (1.03)	.12	27, 8
Gets distracted by social media at work	180, 93/87	2.37 (1.10)	2.63 (1.15)	2.09 (0.98)	.001	27, 18
Feels they get enough sleep	183, 93/90	2.96 (1.11)	2.83 (1.12)	3.09 (1.01)	.10	27, 19
Doesn't have enough time for family	173, 93/80	2.97 (1.08)	3.26 (1.03)	2.63 (1.04)	<.001	28, 8
Considered leaving employment within past year	178, 93/85	2.59 (1.32)	2.69 (1.34)	2.48 (1.30)	.33	28, 3
Considered leaving medicine within past year	182, 93/89	2.65 (1.25)	1.87 (1.14)	1.97 (1.22)	.68	28, 1

Abbreviations: N=number; SD=standard deviation; UC=Underage children living at home

[a] Statistical difference between those with underage children living at home and those without

[b] Indicates the question number of the Likert battery followed by the number of the question in the battery. The survey is provided in Supplemental materials.

Supplemental Table 8: Attitudes and opinions on a 5-point Likert scale: Research. Average agreement with the following statements (1=strongly disagree; 5=strongly agree).

**Radiologists with and without underage children living at home**	N	All	Has UC	Has no UC	*P*-value [a]	Survey question [b]
Statement	All, has UC / has no UC	Mean (SD)	Mean (SD)	Mean (SD)		
Wants to conduct medical research	180, 93/87	3.06 (1.40)	3.13 (1.36)	2.99 (1.44)	.51	27, 9
Feels research is important for career	179, 93/86	2.82 (1.27)	2.91 (1.15)	2.72 (1.39)	.23	27, 11
Inspired by colleagues' research work	181, 93/88	3.52 (1.11)	3.59 (1.08)	3.45 (1.14)	.40	27, 12
Has skills to do research	177, 92/85	3.34 (1.15)	3.42 (1.07)	3.25 (1.23)	.40	27, 13

Feels training for research is available	145, 80/65	2.97 (1.03)	3.10 (0.98)	2.82 (1.09)	.10	27, 14
Able to collaborate in research	154, 85/69	3.04 (1.22)	3.08 (1.18)	2.99 (1.28)	.68	27, 15
Able to attain funding	143, 76/67	3.03 (1.00)	3.07 (0.93)	3.00 (1.09)	.82	27, 10
Passed up research due to lack of time	149, 78/71	3.25 (1.29)	3.44 (1.25)	3.04 (1.31)	.06	28, 4
Passed up research because of family	162, 86/76	2.75 (1.29)	3.02 (1.23)	2.45 (1.29)	.004	28, 7
Not suited for academic research	177, 92/85	2.59 (1.17)	2.57 (1.11)	2.62 (1.23)	.94	28, 5
Considered quitting research (past year)	110, 59/51	2.59 (1.32)	2.71 (1.10)	2.59 (1.40)	.45	28, 2
Does not get along with academic people	174, 91/83	1.84 (0.92)	1.71 (0.79)	1.98 (1.04)	.13	28, 6
Interested in teaching	184, 94/90	3.82 (0.94)	3.79 (0.96)	3.84 (1.02)	.55	27, 2

Abbreviations: N=number; SD=standard deviation; UC=Underage children living at home

[a] Statistical difference between those with underage children living at home and those without

[b] Indicates the question number of the Likert battery followed by the number of the question in the battery. The survey is provided in Supplemental materials.

Supplemental figures

Including:

Study respondents' listed experiences with different types of research

Study respondents' listed sources of research funding between one and five years

Supplemental Figure 1: Experience with different research types (N=134 respondents). The respondents were allowed to choose more than one funding source. Reported by number and percentage.

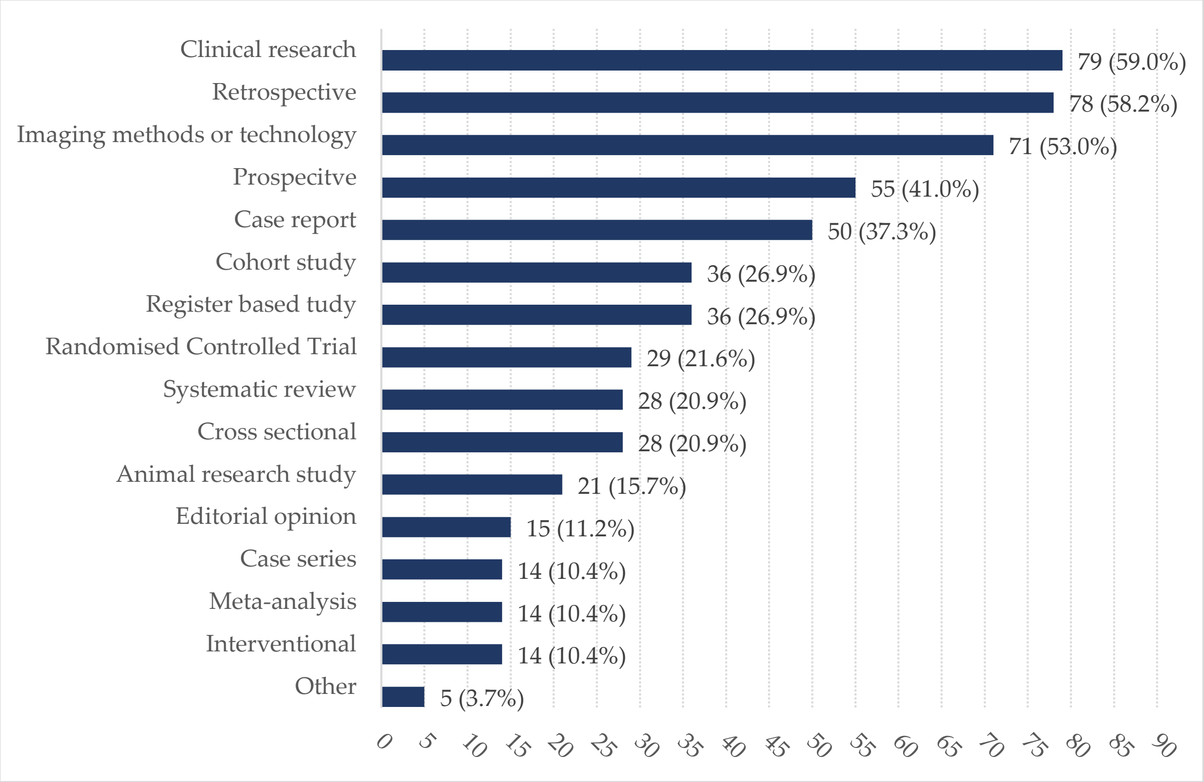

Supplemental Figure 2: Reported funding sources within the past one and five years reported by respondents with research experience (N=134). The respondents were allowed to report multiple funding sources. The number of responses and percentage shares are presented.

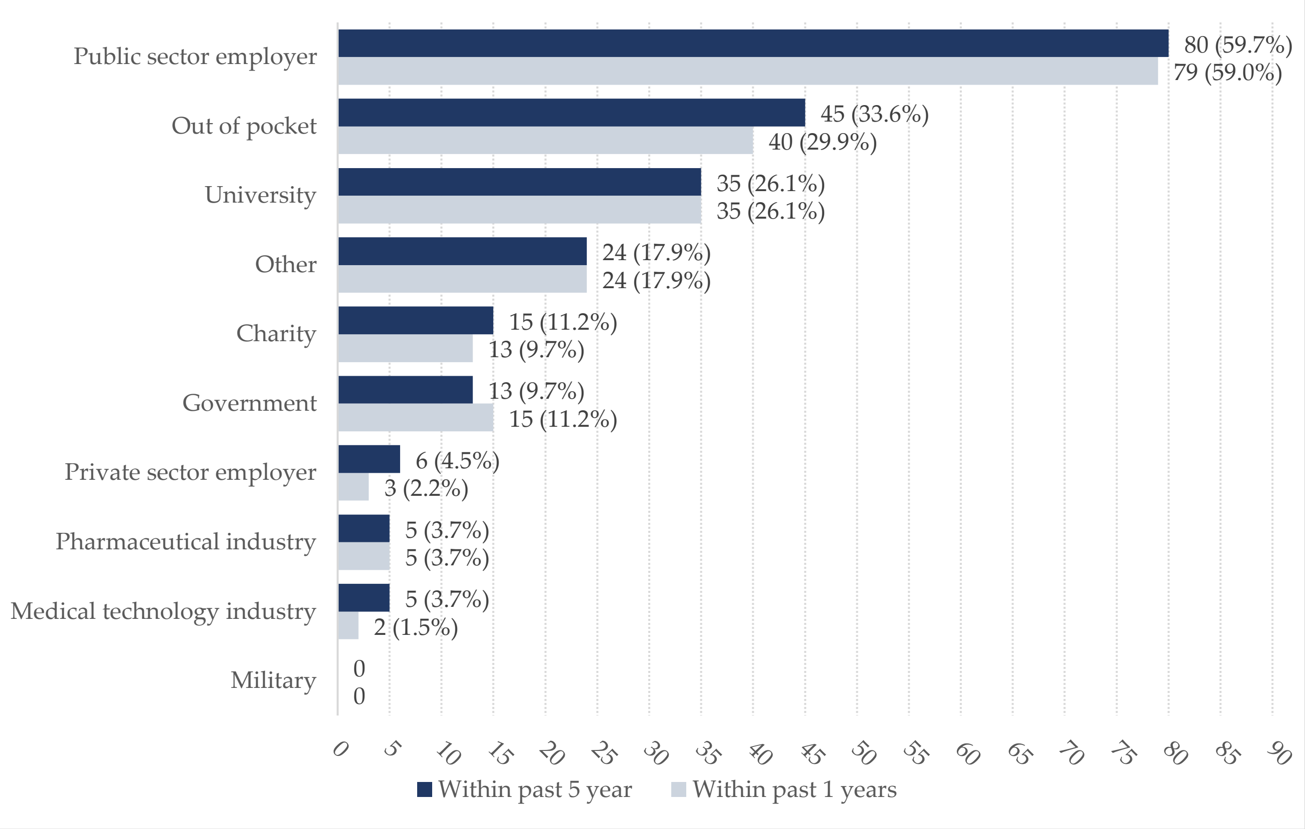

Supplement: Supplementary file 1 — ELECTRONIC SUPPLEMENTARY MATERIAL [file 13244_2025_2108_MOESM1_ESM.pdf]
